# Efficient Near-Field Radiofrequency Imaging of Impact Damage on CFRP Materials with Learning-Based Compressed Sensing

**DOI:** 10.3390/ma15175874

**Published:** 2022-08-25

**Authors:** Huadong Song, Zijun Wang, Yanli Zeng, Xiaoting Guo, Chaoqing Tang

**Affiliations:** 1 SINOMACH Sensing Technology Co., Ltd., Shenyang 110043, China; 2 School of Artificial Intelligence and Automation, Huazhong University of Science and Technology (HUST), Wuhan 430074, China; 3 China Belt and Road Joint Laboratory on Measurement and Control Technology, Wuhan 430074, China

**Keywords:** non-destructive testing, near-field radiofrequency imaging, compressed sensing, deep learning

## Abstract

Carbon fiber-reinforced polymer (CFRP) is a widely-used composite material that is vulnerable to impact damage. Light impact damages destroy the inner structure but barely show obvious change on the surface. As a non-contact and high-resolution method to detect subsurface and inner defect, near-field radiofrequency imaging (NRI) suffers from high imaging times. Although some existing works use compressed sensing (CS) for a faster measurement, the corresponding CS reconstruction time remains high. This paper proposes a deep learning-based CS method for fast NRI, this plugin method decreases the measurement time by one order of magnitude without hardware modification and achieves real-time imaging during CS reconstruction. A special 0/1-Bernoulli measurement matrix is designed for sensor scanning firstly, and an interpretable neural network-based CS reconstruction method is proposed. Besides real-time reconstruction, the proposed learning-based reconstruction method can further reduce the required data thus reducing measurement time more than existing CS methods. Under the same imaging quality, experimental results in an NRI system show the proposed method is 20 times faster than traditional raster scan and existing CS reconstruction methods, and the required data is reduced by more than 90% than existing CS reconstruction methods.

## 1. Introduction

**Figure 1:**
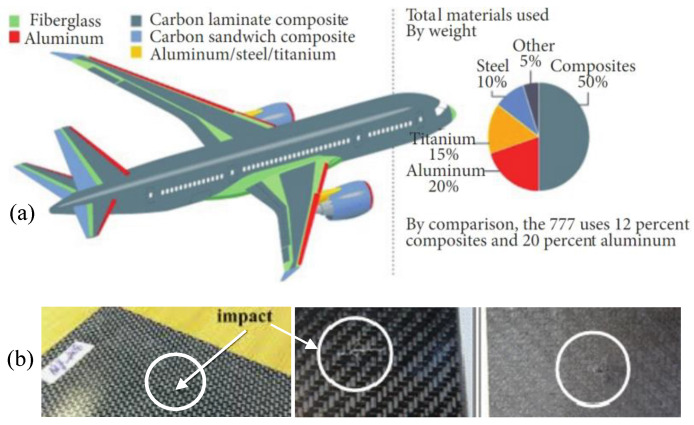
Demo of CFRP application and impact damages. (**a**) Materials used in a Boeing 787 body [2]; (**b**) The barely visible impact damages [5,6].

Carbon-fiber reinforced polymer is a widely-used composite material that uses carbon fiber as the reinforcement element. It usually involves carbon matrices, polymer matrices, metal matrices, or ceramic matrices. CFRP has an excellent strength-to-weight ratio and corrosion resistance, these properties make CFRP popular in the aerospace industry [1]. It is estimated that up to 50% of a Boeing 787 body is made from CFRPs [2]. However, CFRPs are vulnerable to impact forces due to a lack of vertical reinforcement [3], thus resulting in impact damage that greatly reduces the material strength. Light impact forces cause impact damage like disbanding, micro-cracking, and delamination but are barely visible from the surface [4,5,6], as is shown in Figure 1. Therefore, non-destructive testing and evaluation (NDT&E) techniques are required to test the material integrity. 

Among all NDT&E techniques, near-field radiofrequency imaging (NRI) is a non-contact and high-resolution technique based on microwave radio frequency. Compared to X-ray [7], which also enables inner structure detection, NRI does not have the hazardous radiation leakage problem because microwaves are safe for humans. Therefore NRI attracts increasing attention for damage detection on composite materials [8,9,10,11]. Ni et al. [12], propose an NRI-based method called EMW-NDT which shows good detection sensitivity to delamination size and thickness. Li et al. [13], designed a microwave cavity resonator sensor with an octagonal cross-section for thickness measurement of coatings on carbon-fiber composites. He et al. [14], use microwaves to detect CFRP-concrete interfacial defects. Furthermore, targeting CFRP-concrete structures, Islam et al. [15] use a microwave dual waveguide sensor. Lei et al. [16], detect internal defects in metal fiber-composite materials using a double-waveguide probe that is loaded with split-ring resonators. For non-metal internal defects detection, Yang et al. [17], propose a near-field Bessel–Gauss antenna which shows a good performance on composites. Various radio frequencies as used for impact damage detection on CFRPs. Li et al. [18], detect impact damage in carbon fiber composites using an electromagnetic sensor. Salski et al. [19] use printable radiofrequency (RF) inductive sensors that operate on 10~300 MHz to detect delamination, cracks, and voids in CFRPs with a raster scan. K-band frequency [20,21], is used to study microwave responses of impact damages on layered woven CFRP composites. Yang et al. [22], use a 65~67 GHz millimeter wave to successfully detect impact damage with 9 J of impact energy on CFRP. Li et al. [23], use an X-band microwave to characterize and analyze CFRP. Dong et al. [24], use a THz frequency to detect low-velocity impact on hybrid fiber-reinforced composite laminate. Navagato et al. [25], apply microwave noiselets for nondestructive testing of unidirectional carbon fiber-reinforced polymers under ultra-wide bands. These existing works use a raster scan to measure the RF reflectivity of the target area, this is a time-consuming process with a small step-size or large detection area. Salski et al. [19], show that scanning an area of 60 × 200 mm^2^ with eight parallel RF sensors will take about 30 min with 1 mm of step-size. Using more sensors can reduce the measurement time, so large-scale wireless impact monitoring sensors are used to localize the impact damage [26]. 

The hardware-based efficiency improvement methods will increase the cost with more sensors, an alternative is using compressed sensing (CS) to reduce the acquisition points without a hardware update. Tang et al. [27,28,29], propose CS based methods on NRI systems for impact damage detection on CFRPs which greatly reduces the measurement time. CS [30,31], reduces the acquisition data greatly by measuring the linear weightings of an imaging scene. This down-sampling ability is attractive for applications that are sensitive to acquisition time like medical imaging [32], and synthetic aperture radar tomography [33]. Due to indirect measurement, a CS reconstruction process that aims to solve underdetermined equations is needed to obtain the final images. This reconstruction process is also highly time consuming. It takes more than 10 min to reconstruct an image of size 256 × 256 pixels in a block-by-block manner [34,35], a full-size reconstruction takes much longer. This reconstruction process of CS will drag the time gain in the measurement process down.

To achieve truly efficient impact damage detection on CFRP materials using NRI, this paper proposes a learning-based CS method. Artificial intelligence has already gained some attention in the microwave nondestructive testing community [36], most works use it for feature extraction and few works consider the imaging process. Compared to existing CS methods in NRI, the proposed method improves efficiency greatly in both sampling and reconstruction while maintaining the same image quality. This is a totally plugin method that does not need hardware updates. The major contributions are:

- This paper brings in a learning-based CS method for CFRP impact damage detection in NRI, which is 20 times faster than existing methods under the same imaging quality.
- The proposed learning-based CS method brings in a de-nosing ability during RF imaging, which can remove incorrect data in scanning that is extremely hard for traditional methods.
- Instead of being in a black-box as existing deep learning-based CS reconstruction is, the results of the proposed learning-based CS can be anticipated, which is more reliable for sensing applications.
- The proposed method is a plugin method which does not need hardware modification and can be extended to other scanning-based characterization systems.

The rest of this paper is organized as follows: Section 2 introduces the theoretical basis of NRI for impact damage detection on CFRP materials. Section 3 gives the proposed learning-based CS in detail. Experimental results and discussions are presented in Section 4. The final section concludes this paper and suggests some extension works.

## 2. Theoretical Basis of NRI for Impact Damage Detection on CFRP Materials

### 2.1. Near-Field Radiofrequency Imaging for NDT

**Figure 2:**
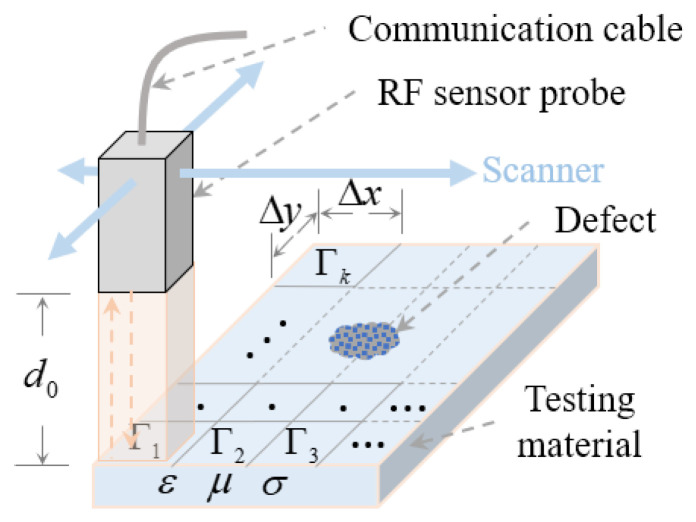
Diagram of a NRI system for NDT.

As is shown in Figure 2, traditional NRI uses a raster scan with step-size Δx and Δy in X and Y directions, respectively, the RF sensor probe emits and receives RF waves in different time slots with a small lift-off distance *d*_0_. The probe can be RF sensors, antennas, waveguides, and etc. An open-ended rectangular waveguide (ORWG) probe with inner dimension a×b is used in this study. The emitted RF waves penetrate the testing material for a distance δ that follows the skin effect equation:

(1) $$\delta =\frac{1}{\sqrt{\pi f\mu \sigma }}$$

where *f* is the operating frequency and μ and σ are the magnetic permeability and electrical conductivity of testing material, respectively. Therefore, a higher frequency has a smaller penetration depth. The received reflection coefficients are complex values Γ which can be modeled as Equation (2) according to transmission line theory:

(2) $$\Gamma =\frac{Z_{in}-Z_{WG}}{Z_{in}+Z_{WG}}$$

where *Z_in_* and *Z_WG_* are the intrinsic impedance of testing material and the waveguide impedance, respectively. The angle between the electrical field vector direction of electromagnetic waves and the fiber direction near the CFRP surface influences Γ. Γ can be easily distinguished from within and outside of the damage areas when the angle is zero, because CFRP is anisotropic material. On the other hand, impact forces change either μ, σ, or permittivity (ε) of the testing material, which changes *Z_in_* as a result. These theoretic bases tell that some frequencies reveal the impact damage better and these frequencies are determined by CFRP structure and properties. Therefore, RF sensors usually work in a frequency-sweeping mode in order to get a wide band resonance.

### 2.2. The CFRP Materials for Case Studies

**Figure 3:**
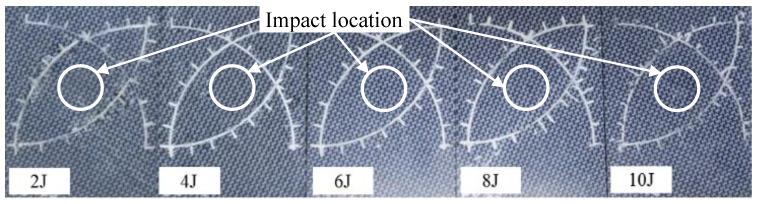
The CFRP specimens in this study.

This study constructed some light impact damage specimens with CFRP materials which were also used in our previous study [27]. The CFRP material in this study is made of polyphenylene sulphide and a thermoplastic resin system. It has 12 layers of 5H satin balanced carbon fiber woven fabrics structure, manufactured by TenCate Advance Composites, Netherlands. Specimens are in a rectangular shape with a size of 100 × 130 mm^2^ and with 3.78 ± 0.05 mm of average thickness. Impact energies ranging from 2 J to 10 J with a 2 J step-size are imposed on different specimens to make light impact damages. Impact energies are controlled by adjusting the height of a free-falling hammer that has a hemispherical bumper head with 20 mm diameter. Figure 3 shows the five specimens, the white scales on each specimen are used for deciding the impact center point. It is obvious that these impact damages are barely visible.

## 3. The Proposed Learning-Based Compressed Sensing

### 3.1. Compressed Sensing Theory and Measurement Matrix Design in NRI

The basic CS theory is remarked upon here briefly. If a vector signal $x\in {\mathbb{R}}^{n\times 1}$ can be represented as a small part of weighted column of a matrix $\Psi \in {\mathbb{R}}^{n\times n}$ as x=Ψs, under CS framework, the signal can be compressively measured with a matrix $\Phi \in {\mathbb{R}}^{m\times n}$ as:

(3) y=Φx+ξ=ΦΨs+ξ=As+ξ

where $y\in {\mathbb{R}}^{m\times 1}$ is the measurement data, Ψ is sparse basis, the weighting vector **s** is called sparse coefficients, Φ is the measurement matrix, and $A=\Phi \Psi \in {\mathbb{R}}^{m\times n}$, $\xi \in {\mathbb{R}}^{m\times 1}$ is the additive measurement noise. The measured signal **y** is a transformed version of the original signal **x**, and is compressed greatly due to  m≪n. Normally, Ψ and Φ are known conditions so **x** can be indirectly obtained by getting the sparse coefficients **s** firstly. Recovering **s** is a typical sparse reconstruction problem:

(4) $$\underset{s}{\min}\;{\left\|s\right\|}_{0}\;\mathrm{subject}\;\mathrm{to}\;{\left\|y-As\right\|}_{2}\leq e$$

where ${\left\|\cdot \right\|}_{0}$ is the $L_{0}$-norm, which means the number of non-zero elements; ${\left\|\cdot \right\|}_{2}$ is $L_{2}$-norm; and *e* is a residual tolerance. There are some optimization theory-based methods like orthogonal matching pursuit (OMP) [37], and iterative hard thresholding [38], to obtain **s**. However, these optimization theory-based methods are time consuming. In recent years, deep learning has obtained attractions in solving this time-consuming reconstruction problem and shows groundbreaking performance [39,40,41,42], but existing deep learning-based methods are barely used in sensing applications due to poor reliability. This paper applies our recently proposed deep learning-based CS reconstruction method called RootsNet [43], to solve this reconstruction problem. RootsNet is much more reliable than existing deep learning methods while inheriting the efficiency benefit.

**Figure 4:**
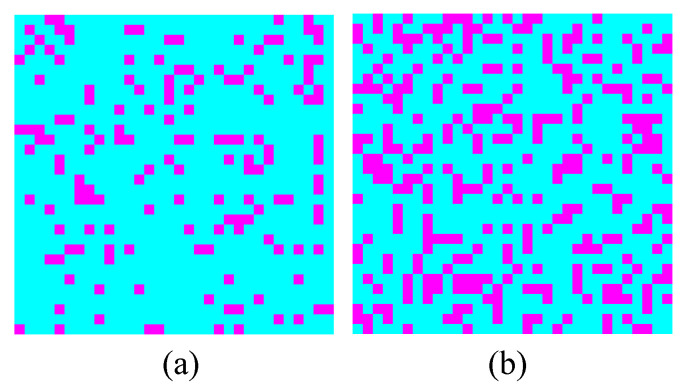
Demo sampling locations (The red dots) on a 32 × 32 area with sampling rates of (**a**) 15% and (**b**) 30% by the designed Φ.

In NRI systems, the RF reflection coefficients obtained by raster scan can be represented as an image **I**. Under CS framework, matrix **I** can be flattened to a vector, i.e., **x**. To enjoy the down-sampling ability of CS, a measurement matrix that fits the hardware ability of NRI systems is necessary. It is proved that 0/1-Bernoulli matrixes [44], and Guassian matrices [45], guarantee sparse recovery in most cases. This paper designs a special 0/1 Bernoulli matrix as shown in Algorithm 1, this new measurement matrix does not require a hardware update for NRI systems. The sampling rate (SR) is *m*/*n*. With this design, there are only *m* numbers of ‘1’ in *m* × *n* elements of the measurement matrix. Each ‘1’ locates in a unique column. During CS measurement in NRI systems, each ‘1’ corresponds to a sampling location in the scanning area, i.e., a single pixel in the final image. Therefore, the designed measurement matrix turns a point-by-point raster scan to a randomly partial scan as is shown in Figure 4, which greatly reduces the measurement time.
**Algorithm 1.** Measurement matrix for NRI systems.
**Input:***m*, *n* **Initial:**$\Phi =0^{m\times n}$, **m** = 1 to *m*;   **n** = random permutate 1 to *n* **Iteration**
*I* = 1 to *m* with step-size 1:   $\Phi_{m(i),n(i)}=1$ **Output:**
Φ

### 3.2. The Proposed Deep Learning-Based CS Reconstruction

**Figure 5:**
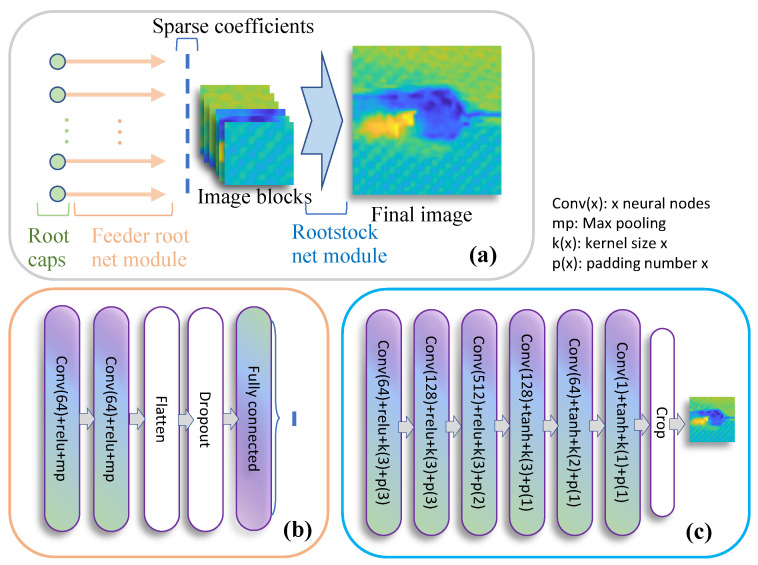
The structure of RootsNet. (**a**) The overall structure; (**b**) The feeder root net module; (**c**) The rootstock net module.

Figure 5 shows the overall structure of RootsNet, which consists of three major parts, i.e., root caps, feeder root net, and rootstock net. Firstly, the measurement **y** and each column of **A** are reshaped into two images with sizes $\sqrt{m}\times \sqrt{m}$. To make $\sqrt{m}$ an integer, a virtual *m* that meets the largest sampling rate is chosen, lower sampling rates are padded with zeros to make *m* constant for different sampling rates. For example, if *n* = 1024, the virtual m is set as 361, which corresponds to the largest sampling rate of *m*/*n* = 0.353 and lets $\sqrt{m}=19$. The two images are concatenated to an image set with size 2 × 19 × 19 which serves as a root cap.

Feeder root net takes root cap as input. Two convolution and max pooling layer blocks are used for feature extraction. The extracted features are flattened to a vector, some of the features are randomly dropped to make the learned feature more robust. Finally, a fully connected layer is used to obtain the corresponding sparse coefficient for each column of **A**. Multiple feeder root branches are used to obtain the sparse coefficients. NRI images are smooth, which means most of the sparse coefficients are in the low frequency range using discrete cosine transform (DCT) as a sparse basis, and the position for large coefficient values are generally constant. Therefore, for *K* feeder root branches, this paper predicts the largest *K* sparse coefficients. Rootstock net takes a fully reconstructed image from feeder root net as input for denoising and deblocking. This is a fully convolutional structure as is shown in Figure 5. Rootstock net and feeder root net can be trained separately.

Compared to existing deep learning-based CS reconstruction methods, RootsNet is more reliable for sensing applications. This is due to the fact that the feeder root branches and sparse coefficients are strictly mapped in a one-to-one manner. The prediction results of feeder root net are sparse coefficients, for common sparse bases like DCT and DWT, the sparse coefficients have a clear physical meaning. For example, sparse coefficients for a DCT basis means the weighting of different frequency components. Therefore, an incorrect prediction only influences the weighting of a frequency component. The distortion on the final image can be anticipated. Existing deep learning-based methods do not have this feature. It should be noted that the proposed learning-based CS method also has over-fitting risk as all deep learning methods. To reduce the effect of overfitting, RootsNet uses one dropout layer to make the learned feature more robust. As a general solution, more training data is also helpful to reduce overfitting.

## 4. Experimental Results & Discussions

To prove the efficiency and imaging quality of the proposed leaning-based CS method, this section carries out experiments in a light impact damage detection problem. The testing specimens are shown in Figure 3. More experimental settings are introduced in Section 4.1.

### 4.1. Settings

#### 4.1.1. Implementation Steps

**Figure 6:**
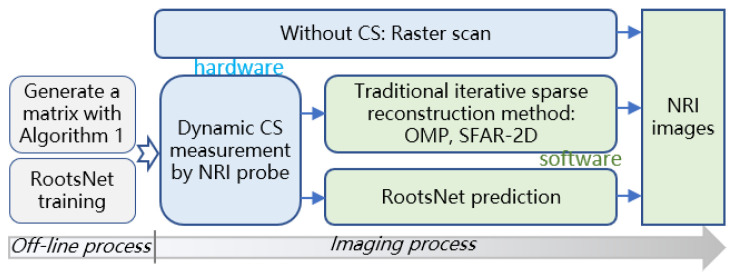
Implementation steps.

The implementation steps are shown in Figure 6 to guide real applications. Traditional raster scan obtains the NRI images directly, CS measurement is a computational imaging way. The proposed RootsNet requires two off-line processes that only need to be done once and can be done off-line before measurement tasks.

For all CS methods, a measurement matrix that corresponds to the largest possible MR (denoted as **M**_m_) is generated using Algorithm 1. Due to the special design of the measurement matrix (which is a sub-sample of the scanning points), different subsets of **M**_m_ can be extracted as measurement matrices that correspond to new MRs. For example, **M**_1_, **M**_2_, and **M**_3_ are corresponding to 3%, 9%, and 15% of MRs, respectively, and **M**_1_ is a subset of **M**_2_, **M**_2_ is a subset of **M**_3_, **M**_3_ is a subset of **M**_m_. In such an implementation, the CS scanning can stop at any time before covering all the scanning locations in **M**_m_, which leads to continuous MRs and a dynamic measurement process. All the measurement matrices are used for RootsNet training before measurement. Both traditional iterative sparse reconstruction methods and the proposed RootsNet are used for NRI image reconstruction using the CS data.

#### 4.1.2. Experimental Setups

**Figure 7:**
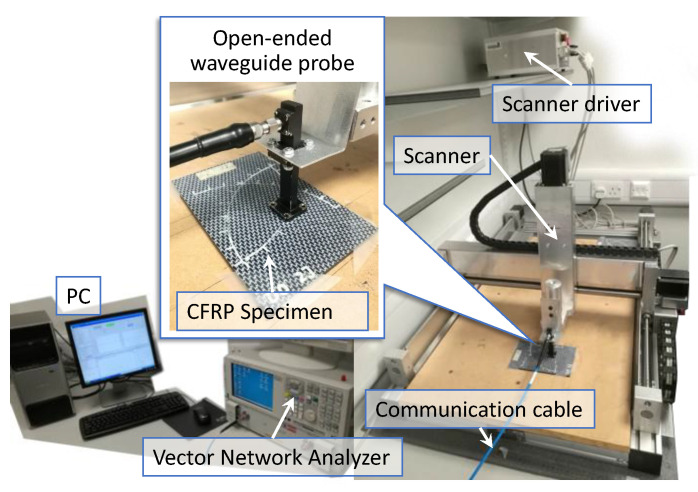
Experimental setups for light impact damage detection on CFRPs.

The overall experimental setup is shown in Figure 7. An XYZ scanner carries a rectangular open-ended waveguide probe which has an inner dimension of 10.668 mm × 4.318 mm for RF emitting and sensing. The RF acquisition signals are generated and captured by a vector network analyzer (Agilent PNA E8363B). A personal computer that connects to the scanner driver is used to control the scanning with a Matlab and general purpose interface bus (GPIB) interface. The frequency sweeping band is set from 18 GHz to 26.5 GHz with 1601 frequency points. The lift-off distance is set as 1.0 mm. The scanning area is a rectangular shape with the size 30 mm × 30 mm that covers the impact center. Step-size in both X and Y directions are set as 0.3 mm. Accordingly, the image size is 99 × 99. To fit with the learning method, we limit the scanning point to 96 × 96 that can be fully divided by a block size of 32. This force limitation is not compulsory because an image block can pad zeros without exact division. All five specimens are scanned in sequence as a reference result.

#### 4.1.3. Configuration for Neural-Network Training

Existing works [27,28], show that there are some resonant frequency bands that review impact damages better, i.e., 18.86 GHz, 20.65 GHz, and etc. The amplitudes of reflection coefficients are used for imaging. The detailed neural network structures are shown in Figure 5. To train the neural network model, 50 images within the resonant frequency bands for each specimen are picked out as a training set, which makes up a dataset that consists of 250 images. The block size of the set is 32, so there are 2250 image blocks in total, they are divided randomly into a training set and a validation set with a ratio of 8:2. Equivalent CS sampling is applied on raster scan data during model training. Different sampling rates that range from 3% to 33% with an equal step-size of 6% are considered. It should be noted that the sampling rate is almost linearly proportional to sampling time in NRI systems, i.e., a 3% sampling rate will reduce around 97% of sampling time than a traditional raster scan. 

The feeder root net and rootstock net are trained separately. Neural network models are trained on a desktop computer with a RTX3090 GPU and a Core-i9 10900K CPU on an open-source deep learning framework, Paddlepaddle. The learning rates are initiated as 0.01 and gradually decay to 1 × 10^−5^ during the 1500 epoch of training. Feeder root nets use mean square error (MSE) as a loss function between the predicted sparse coefficients and ground truth coefficients. Ground truth coefficients are obtained by discrete cosine transform on flattened image blocks. As is discussed in Section 3.2, this paper builds 256 feeder root net branches to recover the most significant 256 coefficients. Rootstock net uses structure similarity (SSIM) between a ground truth image and the predicted image as loss function. After training, the same measurement matrices are used for testing.

### 4.2. Accuracy Analysis

**Figure 8:**
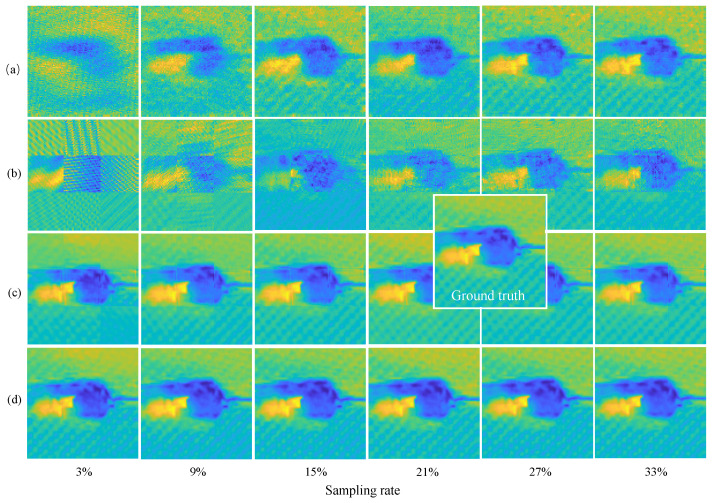
Imaging results on the 10 J specimen. (**a**) The OMP algorithm; (**b**) Block-by-block reconstruction with OMP; (**c**) the proposed feeder root net output; (**d**) Final output for the proposed RootsNet.

**Figure 9:**
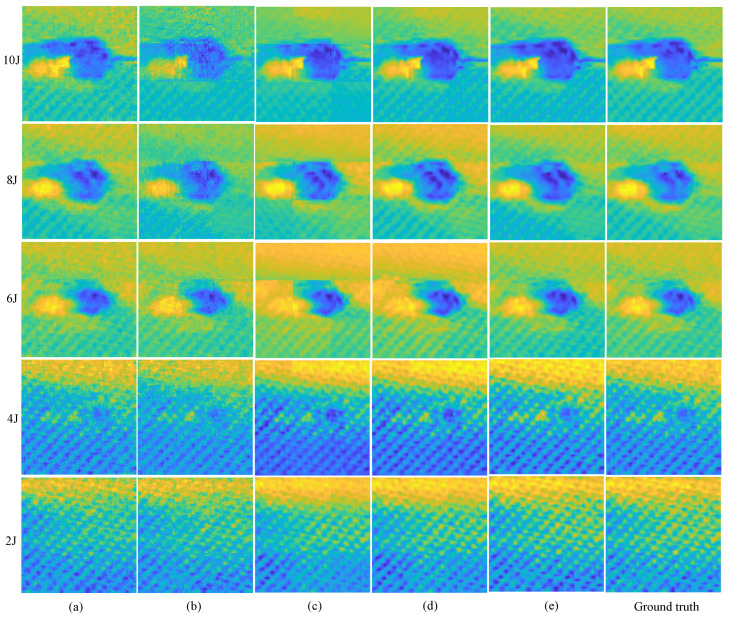
Imaging results on different specimens. (**a**) OMP with 33% of sampling rate; (**b**) Block OMP with 33% of sampling rate; (**c**) the proposed feeder root net output with 3% of sampling rate; (**d**) the final output of the proposed RootsNet with 3% of sampling rate; (**e**) the final output of the proposed RootsNet with 33% of sampling rate.

The state-of-the-art OMP [27], and block-OMP [29], methods in this field are used as baselines. The raster scan results are used as ground truth. The residual errors are set as 1 × 10^−3^ and 1 × 10^−6^ respectively for OMP and block OMP. Figure 8 shows imaging results on the 10 J specimen for different methods. After using the block reconstruction method, OMP method obtains lower image quality and the block effect is obvious. All methods achieve a better quality with a greater sampling rate, this is more obvious for traditional optimization theory-based methods (e.g., OMP). The proposed RootsNet achieves good image quality even at an extremely low sampling rate. Feeder root net output shows some block effect as well, but not as obvious as OMP. This block effect is removed by rootstock net totally. Figure 9 shows more results, even the worst results of the proposed method (3% of sampling rate) can match with the best results (33% of sampling rate) for the state-of-the-art OMP methods. Generally, the proposed method shows smoother results than OMP because only the most significant 256 among the 1024 coefficients are recovered by RootsNet. OMP obtains some incorrect high frequency components during iteration.

**Figure 10:**
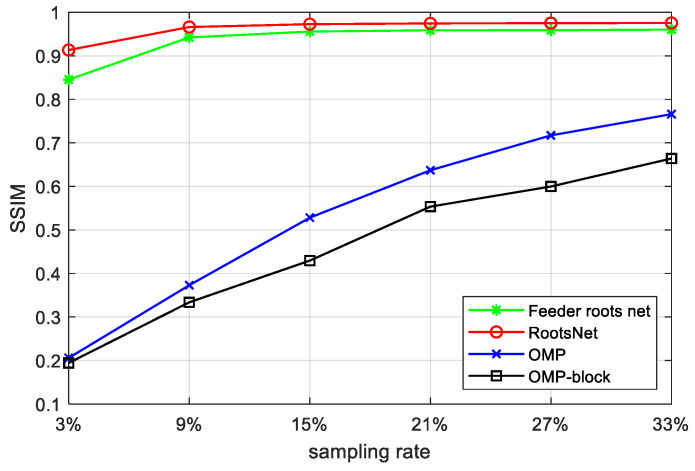
Average SSIM between the reconstruction results of different methods and the ground truth on all specimens.

**Figure 11:**
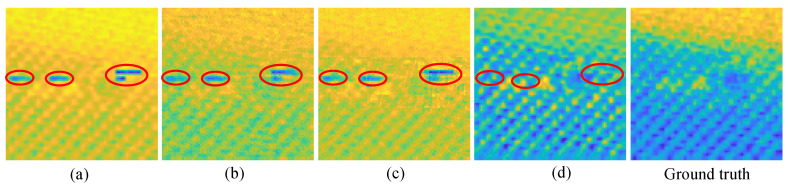
Denoising ability of the proposed RootsNet. (**a**) A raster scan image which has wrong data (highlighted in red circles); (**b**) OMP imaging results; (**c**) block OMP imaging results; (**d**) RootsNet imaging results.

Figure 10 shows quantitative imaging quality results, which shows a great quality gain by the proposed method. The worst results of RootsNet also better than the best results of OMP, which means RootsNet reduces at least (33 − 3)/33 ≈ 91% more of sampling data and reconstruction time than OMP, and has three to four times the quality gain in low sampling rates (4.3 times in 3% and 2.7 times in 9%). Traditional optimization theory-based methods find sparse coefficients with simple rules like correlation calculation, the simple rules are easily corrupted by noise under low sampling rates. Feeder root net extracts high-dimensional features instead, that is why it performs much better. The rootstock net automatically learns some priori information from the training set to remove block effect and wrong data or noise (as is shown in Figure 11). Figure 11 shows the de-nosing ability for the proposed RootsNet. The wrong data in the red circles is likely caused by equipment shake during scanning. It is extremely hard for optimization theory-based methods to recognize and remove such noise data. 

### 4.3. Efficiency Analysis

**Figure 12:**
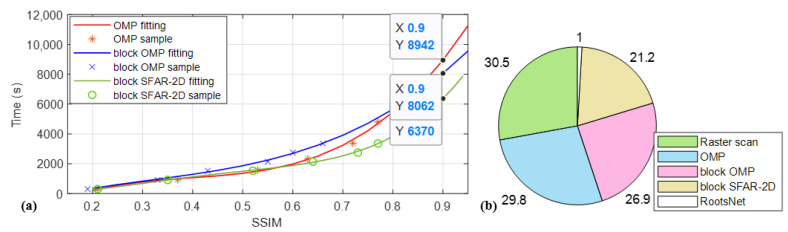
Average time vs. imaging quality. (**a**) The estimated time for traditional iterative reconstruction methods when reaching 0.9 of SSIM by fitting; (**b**) Normalized time consumption of different methods when reaching 0.9 of SSIM.

Compressed sensing saves sampling time greatly but incurs reconstruction time which is a bottleneck for traditional optimization theory-based sparse reconstruction methods. Among all the traditional sparse reconstruction methods, greedy algorithms have a good balance between computational complexity and accuracy. Therefore, this part compares the proposed method with the state-of-the-art greedy algorithms OMP in this field and a recent SFAR-2D [46]. Block reconstruction is used for SFRA-2D to further improve the efficiency. The CS reconstruction times for different methods are given in Table 1. All methods are implemented in Python on the same computer. Without block reconstruction, the reconstruction time of OMP methods increases rapidly. Block reconstruction greatly reduces the reconstruction time but also increases quickly for increased sampling rates for both OMP and SFAR-2D due to the iterative nature. The proposed RootsNet processes image blocks and feeder root net branches parallelly, so the time is almost same for different sampling rates. To see how time consumption versus imaging quality, Table 2 gives the total time consumption and SSIM for different methods. The SSIM of raster scan is defined as the sampling rate because it is a purely down-sampling of the final image before it is finished scanning. Improving imaging quality sacrifices time greatly for all methods due to almost linear increasing between sampling time and sampling rate. A real application needs to balance time and quality. Figure 12b shows the time consumption of different methods when reaching 0.9 of SSIM. The time for OMP methods is estimated by polynomial fitting in Matlab. The long reconstruction time for traditional optimization-based methods counteracts the time saved in CS measurement. In this study, when reaching 0.9 of SSIM, the proposed RootsNet are 30 times faster than traditional raster scan and the-state-of-the art CS methods in this field, OMP. The SFRA-2D is faster than OMP, but also much slower than the proposed RootsNet.

## 5. Conclusions & Future Works

This paper proposes a learning-based CS method for CFRP impact damage detection with near-field radio frequency imaging, which is 20 times faster than existing methods under a same imaging quality. The proposed method brings in de-nosing ability during RF imaging, which can remove incorrect data in scanning that is extremely hard to remove using traditional methods. Instead of being a black-box as existing deep learning-based CS reconstruction, the results of the proposed learning-based CS can be anticipated, which are more reliable for sensing applications. This plugin method does not need hardware modification and can be extended to other scanning-based characterization systems.

There are still some limitations that are worth improving. The proposed method uses pre-defined measurement matrices, which need to be re-trained for a different measurement matrix. Some deterministic matrices may have better performance than Bernoulli matrices [47], which leads to increased data-saving when reaching the same imaging quality. Some convex optimization methods may also lead to more data reduction [48]. On the other hand, although the feeder root net has good interpretability, the rootstock net is an end-to-end method. The next step can consider an inter-block optimization scheme in the feeder root net to maintain the imaging quality and interpretability at the same time. 

## Figures and Tables

**Table 1:** CS reconstruction time (in seconds) for different methods.

	SR	3%	9%	15%	21%	27%	33%
Method							
OMP		0.52	8.74	50.54	196.42	628.57	1445.63
Block OMP		0.09	0.74	1.68	3.63	6.24	11.50
Block SFAR-2D		**0.07**	0.63	1.22	2.92	5.21	8.69
**RootsNet**		0.42	**0.42**	**0.43**	**0.43**	**0.43**	**0.44**

**Table 2:** Total time (in seconds) consumption and SSIM for different methods.

	SR	3%	9%	15%	21%	27%	33%
Method							
Raster scan		304.2/0.03	917.3/0.09	1531.2/0.15	2137.4/0.21	2746.8/0.27	3352.2/0.33
OMP		305.72/0.21	926.04/0.37	1581.74/0.53	2333.82/0.63	3375.37/0.72	4797.83/0.77
Block OMP		304.29/0.19	918.04/0.33	1532.88/0.43	2141.03/0.55	2753.04/0.60	3363.70/0.66
Block SFAR-2D		**304.27**/0.21	917.93/0.35	1532.42/0.52	2140.32/0.64	2752.01/0.73	3360.89/0.77
**RootsNet**		304.62/**0.91**	**917.72/0.96**	**1531.63/0.97**	**2137.83/0.97**	**2747.23/0.97**	**3352.64/0.98**

Note: Time and SSIM are given in ‘time/SSIM’ in the data section.

## Data Availability

The data supporting reported results by the authors can be sent by e-mail.
